# Prenatal diagnosis of congenital megalourethra with imperforate anus

**DOI:** 10.1186/s12887-019-1510-y

**Published:** 2019-04-23

**Authors:** An-Shine Chao, Yao-Lung Chang, Peter Ching-Chang Hsieh

**Affiliations:** 1 Department of Obstetrics and Gynecology, Chang Gung Memorial Hospital and Chang Gung University, LinKou, Republic of China; 2 Department of Obstetrics and Gynecology, Chang Gung Memorial Hospital and Chang Gung University, Taipei, Republic of China; 3 Department of Obstetrics and Gynecology, Chang Gung Memorial Hospital and Chang Gung University, 5, Fu Shin street, Kwei Shan, Tao Yuan, 333 Taiwan

**Keywords:** Megalourethra, Imperforate anus, Prenatal diagnosis

## Abstract

**Background:**

Congenital megalourethra is a rare prenatal finding while prenatal diagnosis of imperforate anus poses high challenge. This is the first prenatally ultrasound diagnosed case which had congenital megalourethra and imperforate anus. This case demonstrated the possibility of using the prenatal imaging findings to evaluate the postnatal prognostic outcomes in multi-organ anomalies.

**Case:**

We present a case of congenital megalourethra, diagnosed prenatally at 22 weeks’ gestation, in which the penis appeared severe dilated with complete absence of the corpora spongiosa and cavernosa. This case also revealed absence of perianal muscle which was in associated with imperforate anus. Detailed prenatal ultrasonographic findings predicted the high possibility of poor outcome of the fetus in the pulmonary, renal, and sexual functions.

**Conclusion:**

This case serves to identify not only the marked bilateral hydronephrosis features but also the striking lower urethral malformation with obstruction flow effect of the penis. Indeed we believe this is the first case report of a rare case of fetal megalourethra associated with imperforate anus at early second trimester on ultrasonography imaging.

## Background

Fetal megalourethra is rare and mostly associated with severe renal dysfunction and oligohydramnios [1, 2] most probably due to a consequences of lower urinary tract obstruction. Congenital megalourethra is characterized by an enlarged penis caused by cystic dilatation of the urethra, resulting from the absence or hypoplasia of the corpus spongiosum or corpus cavernosum or anterior urethral valves [2, 3]. We present the prenatal sonography and postnatal findings of a fetus at 22 weeks of gestation with a marked dilated penis, megacystis, bilateral hydronephrosis and in combined with imperforate anus.

## Case presentation

We report a 30-year-old women, gravida 2, para 1 was referred for an ultrasound examination at 22 weeks gestation for abnormal fetal abdominal dilated cystic lesions. Her family history and prenatal course have been unremarkable.

**Fig. 1:**
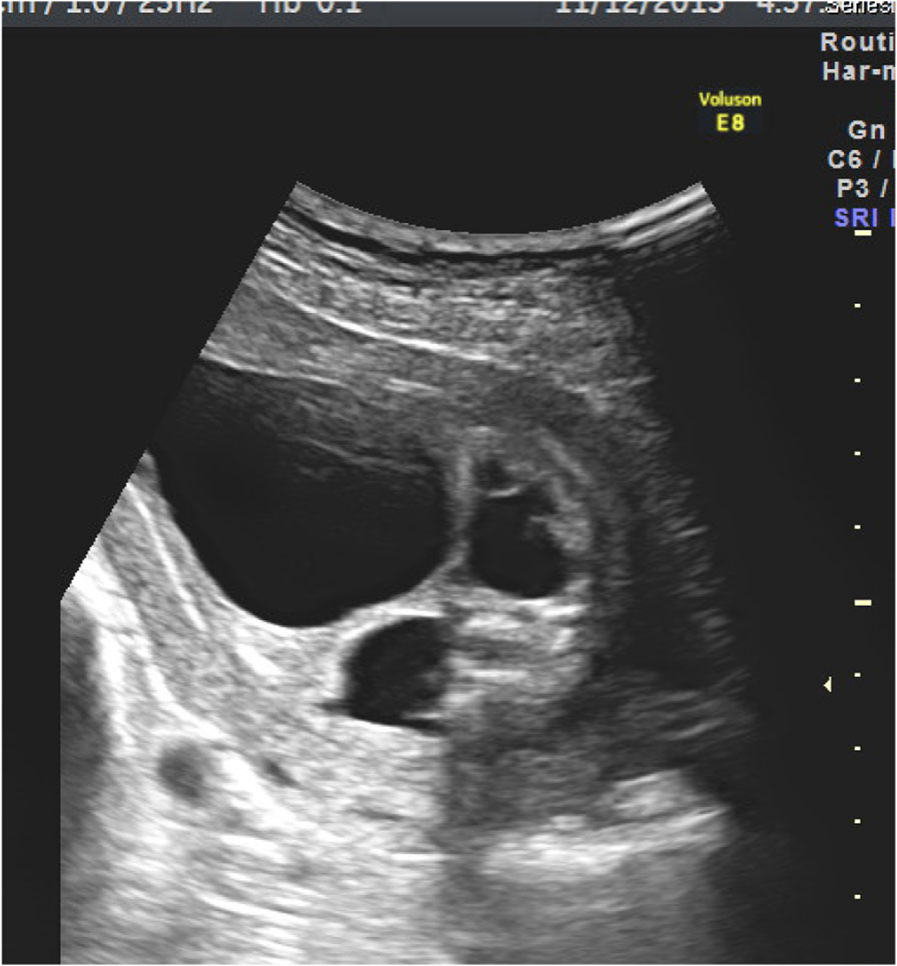
Severe bilateral hydronephrosis and megacystis

**Fig. 2:**
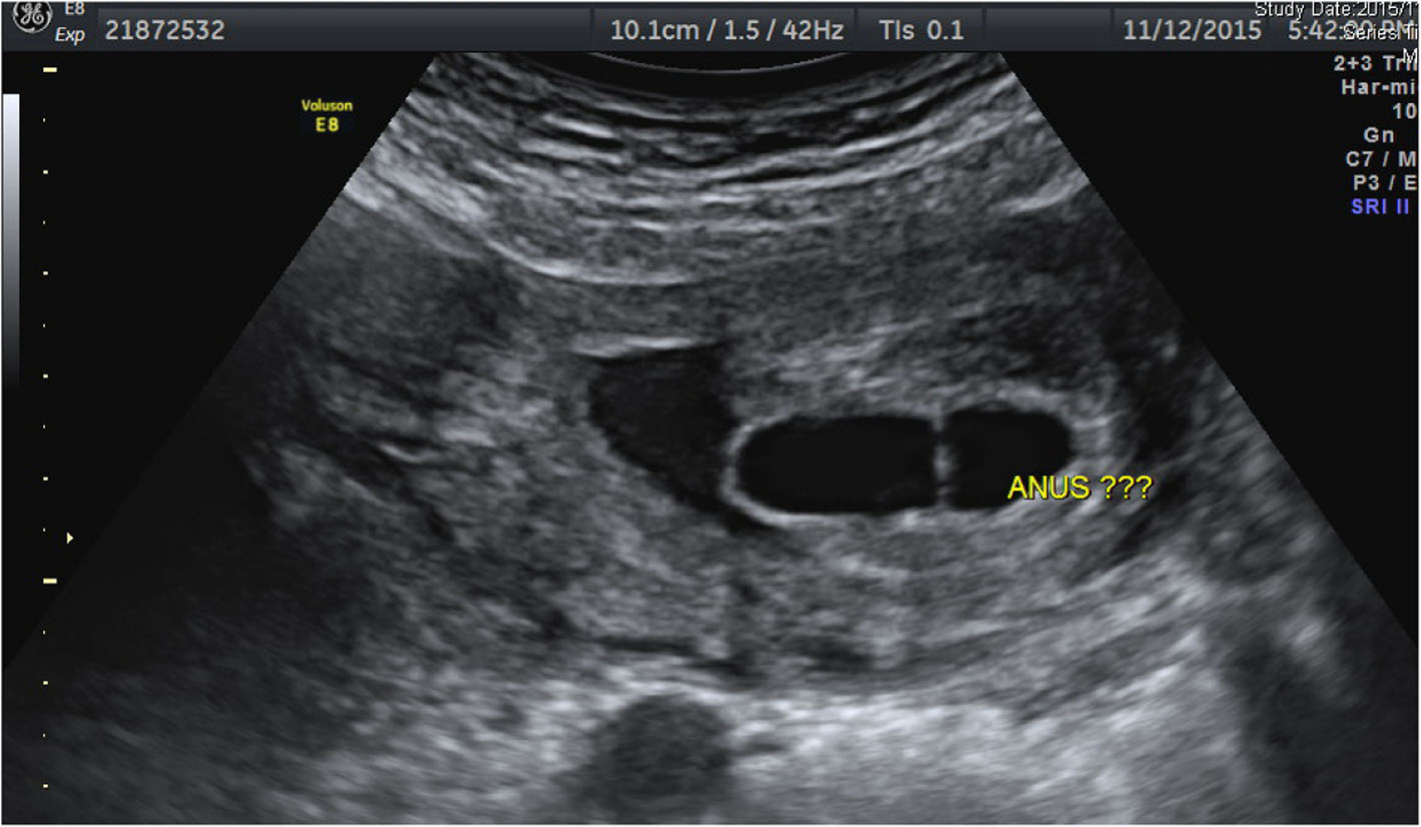
Megalourethra.and imperforate anus. Large cystic change of the penis with no demonstration of the anal sphincter nor the echogenic anal mucosa at the posterior perineal triangle

**Fig. 3:**
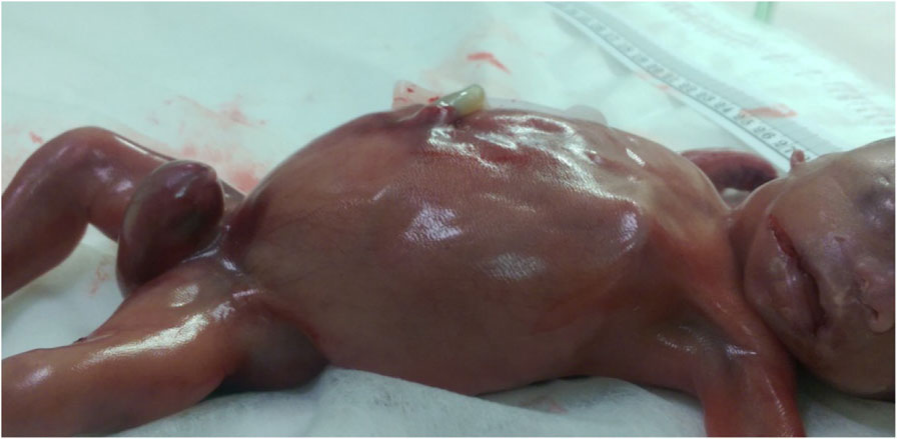
Gross examination of the fetus. In axial image showing: distended abdomen due to severe hydronephrosis and urine retention in bladder, with enlarged dilated penis and imperforate anus

**Fig. 4:**
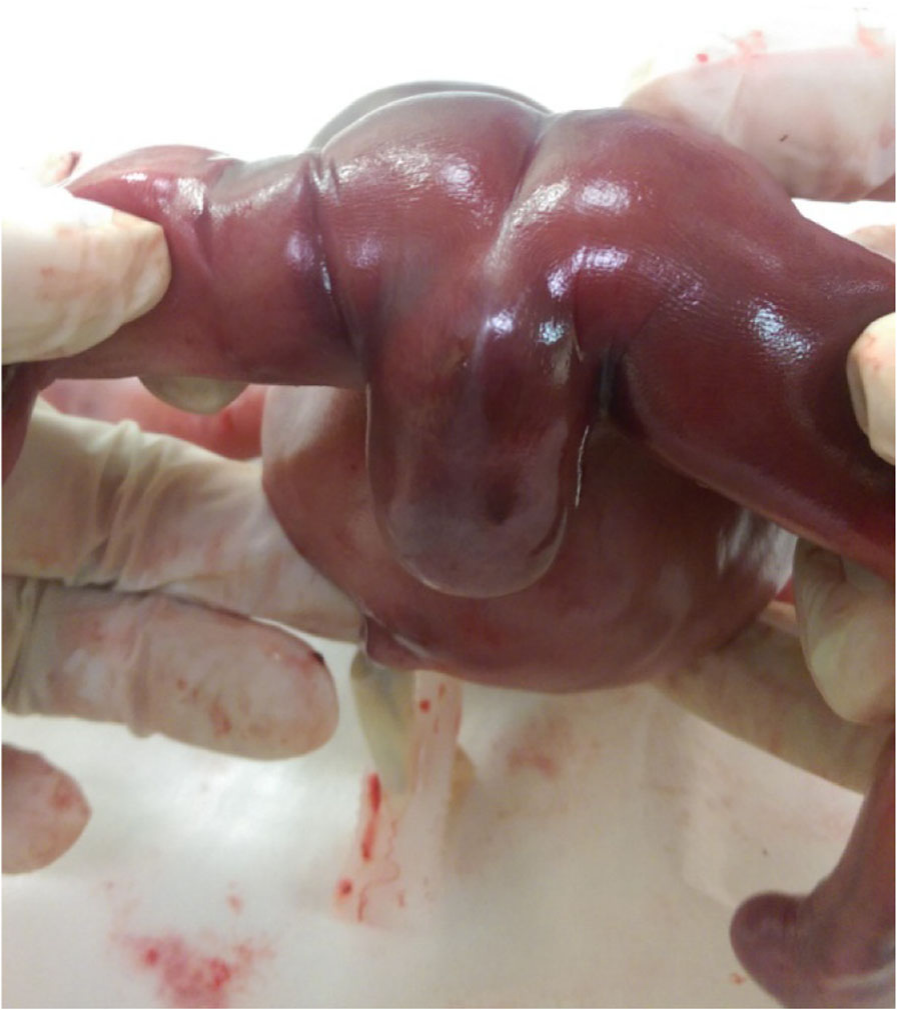
Gross examination of the fetus. In sagittal images showing: distended abdomen due to severe hydronephrosis and urine retention in bladder, with enlarged dilated penis and imperforate anus

The ultrasound evaluation revealed very prominent abnormalities over the entire urology system with marked bilateral hydronephrosis, hydroureters and megacystis, with extension of an enlarged cystic and septate lesion in the penis (Fig. 1). In addition to the urology lesions, the posterior perineum region show absence of the anal muscle in which an anorectal anomaly was highly suspected (Fig. 2). The amniotic fluid was decreased but not yet anhydramnios. Other organ systems did not have detectable ultrasonography anomaly. The parents underwent counseling and decided to terminate this pregnancy because of a poor prognosis mainly caused by the high probability of severe renal and sexual malfunctions. The gross examination of the 465 g fetus confirmed the prenatal diagnosis of megalourethra and imperforate anus (Figs. 3 & 4), however, autopsy and genetic study were declined.

## Discussion & Conclusion

Congenital megalourethra was defined by dilatation and elongation of the penile urethra on prenatal sonography with a remarkable postnatal examination. This is an uncommon male urogenital anomaly with only limited numbers of case reports [1–3].

Among the largest multi-center cohort case collection of fifty cases of congenital megalourethra, none reported prenatal diagnosis of imperforate anus [2, 3]. Most prenatally diagnosed fetuses, nearly 90%, with congenital megalourethra were diagnosed in the second trimester [1–3]. They are usually associated with varies severity of bilateral hydroureters, hydronephrosis and decreased amniotic fluid. In addition, half of the megalourethra had extra-urogenital anomaly [3]. The major pathogenic cause is obstructive of urine flow, which may be complete or partial, is along with consequent renal and pulmonary failure. Only 10% cases, which had no other genitourinary anomalies, had spontaneous resolution with normal postnatal renal function [3]. Our case was postulated as caused by complete absence of the corpora spongiosa and cavernosa, resulting partially obstructed the flow and had associated renal parenchyma damage. Fetal interventions did not affect either the survival rate or renal function.

On the other aspect, imperforate anus has a low prenatal detection rate. It also has a low incidence congenital defect in the general population (0.04%) but has 30 times frequent incidence in high-risk group of congenital anomaly of urinary tract (1.2%) [4]. Early reports of prenatal diagnosis have relied on indirect sonographic findings such as over distention of the rectum and even to the sigmoid colon. Under the application of high resolution ultrasonography, the earliest detection of anal anomaly was by 20 weeks of gestation [4]. This case was consistent with previous reports with absence of the anal mucosa in the posterior perineal triangle in the trans-perineal axial view is a specific and important sonographic marker for improperly canalized anal canal and a direct prenatal diagnosis of imperforate anus.

As the genetic and autopsy studies were unavailable, the true underlying possible pathogenesis of this case such as chromosome aneuploides, megacystis-microcolon hypoperistalsis (MMIHS) and other genetic syndromes, was uncompleted. The genito-recto malformation originates from the endoderm were identified in some animal studies. The defects in sonic hedgehog signaling and the mutations in the homebox genes will hamper proper functions of the urogenital sinus [5]. Furthermore, the outcomes of megalourethra not only depends on the postnatal renal and pulmonary functions but also on voiding and sexual dysfunction [4, 6]. The prenatal detection of a severe megalourethra with hydronephrosis, as in our case, following an addition of anal anomaly yielded a poorer outcome.

The present case revealed a unique finding: early prenatal onset of a large megalourethra and imperforate anus can be evaluated through ultrasonography. We also demonstrated the usefulness of prenatal sonography in a high-risk patient for having both the urogenital and anorectal lesions as well as to evaluate the severity, and prognosis of the postnatal outcomes. Though prenatal diagnosis remains a great challenge, this case proved feasible and will be beneficial experience in optimizing perinatal care and better parents counseling.

## Abbreviation

MMIHS: Megacystis-microcolon hypoperistalsis

## References

[1] Amsalem H, Fitzgerald B, Keating S (2011). Congenital megalourethra: prenatal diagnosis and postnatal/autopsy findings in 10 cases. Ultrasound Obstet Gynecol.

[2] Promsonthi P, Viseshsindh W (2010). Case report and review: prenatal diagnosis of congenital megalourethra. Fetal Diagn Ther.

[3] Moaddab A, Sananes N, Hernandez-Ruano S (2015). Prenatal diagnosis and perinatal outcomes of congenital Megalourethra: a multicenter cohort study and systematic review of the literature. J Ultrasound Med.

[4] Perlman S, Bilik R, Leibovitch L, Katorza E, Achiron R, Gilboa Y (2014). More than a gut feeling – sonographic prenatal diagnosis of imperforate anus in a high-risk population. Prenat Diagn.

[5] Chen Y, Jiang J (2013). Decoding the phosphorylation code in hedgehog signal transduction. Cell Res.

[6] Aulbert W, Kemper MJ (2016). Severe antenatally diagnosed renal disorders: background, prognosis and practical approach. Pediatr Nephrol.

